# Uncovering the Role of Metabolism in Oomycete–Host Interactions Using Genome-Scale Metabolic Models

**DOI:** 10.3389/fmicb.2021.748178

**Published:** 2021-10-11

**Authors:** Sander Y. A. Rodenburg, Michael F. Seidl, Dick de Ridder, Francine Govers

**Affiliations:** ^1^Laboratory of Phytopathology, Wageningen University & Research, Wageningen, Netherlands; ^2^Bioinformatics Group, Wageningen University & Research, Wageningen, Netherlands; ^3^Theoretical Biology & Bioinformatics group, Department of Biology, Utrecht University, Wageningen, Netherlands

**Keywords:** genome-scale metabolic model, systems biology, metabolic networks, *Phytophthora infestans*, plant pathogenic oomycetes, plant–pathogen interactions

## Abstract

Metabolism is the set of biochemical reactions of an organism that enables it to assimilate nutrients from its environment and to generate building blocks for growth and proliferation. It forms a complex network that is intertwined with the many molecular and cellular processes that take place within cells. Systems biology aims to capture the complexity of cells, organisms, or communities by reconstructing models based on information gathered by high-throughput analyses (omics data) and prior knowledge. One type of model is a genome-scale metabolic model (GEM) that allows studying the distributions of metabolic fluxes, i.e., the “mass-flow” through the network of biochemical reactions. GEMs are nowadays widely applied and have been reconstructed for various microbial pathogens, either in a free-living state or in interaction with their hosts, with the aim to gain insight into mechanisms of pathogenicity. In this review, we first introduce the principles of systems biology and GEMs. We then describe how metabolic modeling can contribute to unraveling microbial pathogenesis and host–pathogen interactions, with a specific focus on oomycete plant pathogens and in particular *Phytophthora infestans*. Subsequently, we review achievements obtained so far and identify and discuss potential pitfalls of current models. Finally, we propose a workflow for reconstructing high-quality GEMs and elaborate on the resources needed to advance a system biology approach aimed at untangling the intimate interactions between plants and pathogens.

## Introduction

**Figure 1:**
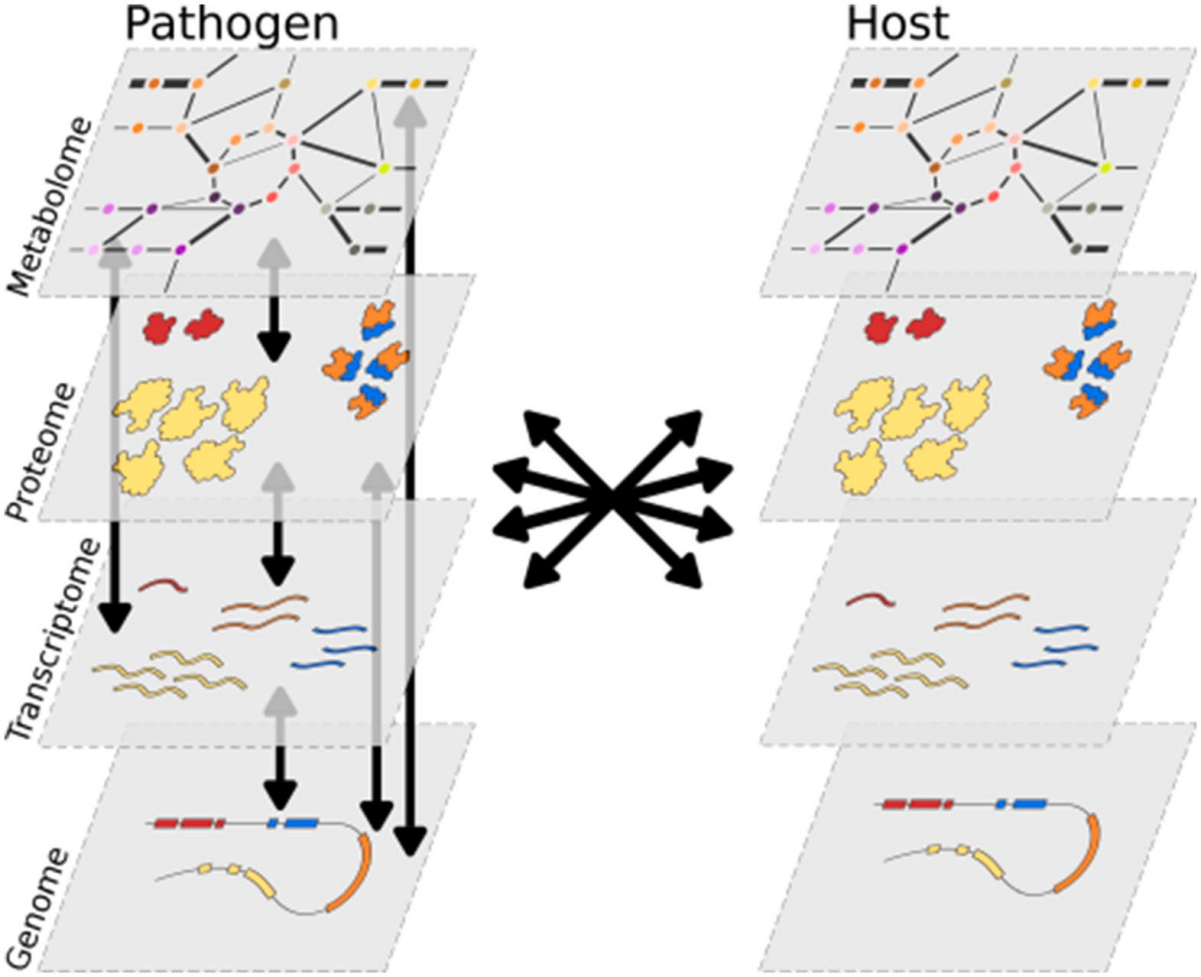
The molecular layers of a cell are all interconnected and form a complex and integrated system. In the symbiosis between a pathogen and a host, their systems are connected, and interactions occur between all molecular layers.

The metabolism of an organism defines its capabilities to take up nutrients from the environment and to convert these into essential building blocks such as nucleic acids and amino acids (Lazar and Birnbaum, 2012). Cellular metabolism can be described as a system of biochemical conversions (reactions), most of which are catalyzed by enzymes. Prokaryotic and eukaryotic genomes can encode hundreds to a few thousand metabolic enzymes (Yilmaz and Walhout, 2017). Each enzyme acts on a selection of substrates and converts these into products, typically by adding or removing reactive groups. Reactions that share substrates or products can be considered functionally connected. The collection of biochemical reactions within a cell thus forms a large, interconnected network that represents the routes by which an organism converts simple nutrients into complex metabolites and vice versa. This network is distributed over different subcellular compartments (organelles), and transporter proteins as well as channels facilitate the transport of metabolites across lipid bilayers that surround the cell and the organelles (Sahoo et al., 2014). The overall system is subject to many parameters, such as variability in substrate concentrations, temperature, or the pH, not only in the extracellular space but also within cells. Cells regulate this system to maintain homeostasis, i.e., the ability to perform important cellular functions despite variations (perturbations), which provides robustness (Eberl, 2018; Nijhout et al., 2018). The ability to sense environmental variations and metabolic cues, and to adapt metabolism accordingly, depends on a tightly interlinked regulatory system that involves feedback loops embedded in interaction networks crossing metabolic, protein, transcript, and (epi) genetic levels (Figure 1; Watson et al., 2015). As such, the phenotype of the cell is an emergent property of the system’s complexity (Aderem, 2005). The rates of individual metabolic reactions are tightly linked to the overall state of cellular metabolism, and therefore, understanding a small part of the system (e.g., a single enzyme or pathway) provides only limited insight into the complete system. Thus, holistic approaches are essential to understand how the state of a system can lead to the complex phenotype of an organism. Systems biology is a discipline based on such holistic approaches.

In this review, we first provide a broad overview of the field of systems biology, with a focus on genome-scale metabolic models (GEMs). We then discuss the relevance of GEMs to study pathogens and host–pathogen interactions, particularly oomycete plant pathogens such as *Phytophthora infestans*. We provide an overview of recent developments in this field and discuss challenges in reconstructing GEMs in these organisms. Finally, we propose a workflow for reconstructing high-quality GEMs and lay out a number of challenges that need to be addressed for systems biology to provide its full potential to study the intimate interactions between plants and pathogens.

## Systems Biology Provides a Holistic Overview

The rise of computational biology, high-throughput analyses tools, and advanced measurement technologies in the early twenty-first century enabled biologists to analyze the presence of basically all molecules in an organism and to measure their quantities and interactions at cell or tissue level (Reed et al., 2006). Today, the genomes of numerous species have been sequenced, and transcriptome sequencing as a proxy for gene expression levels has become routine. Additionally, high-throughput methods such as mass spectrometry allow the analyses of the proteome and metabolome. Data analyses typically focus on differential abundances of transcripts, proteins, or metabolites between samples. Alternatively, omics data can be used to construct models of molecular systems, as part of a scientific discipline known as systems biology (Bordbar et al., 2014) that integrates—among others—bioinformatics, mathematics, biochemistry, molecular biology, physics, and engineering (Breitling, 2010). In systems biology, omics data are integrated with prior experimental knowledge into models of molecular interactions at genome scale, aiming to capture the complete molecular systems that result in the phenotype of an organism (Yurkovich and Palsson, 2016). This includes models of protein–protein interactions, metabolic fluxes, regulatory interactions, or signaling pathways (Albert, 2007). In systems biology, high-throughput data are integrated into computational models that describe the state of the whole system.

## Modeling Metabolism

Many different types of models exist, such as ordinary differential equation (ODE)-based models for enzyme kinetics, Bayesian, Boolean, or rule-based models for signaling and regulatory networks, or constraint-based steady-state models of metabolism such as GEMs; Box 1; Bordbar et al., 2014; Bartocci and Lió, 2016). Metabolism is arguably the best described cellular system, and the availability of reaction information (i.e., the conversions catalyzed by metabolic enzymes) makes GEMs particularly powerful tools to investigate this system and to model metabolic fluxes within an organism (DeBerardinis and Thompson, 2012; Gu et al., 2019). They are based on the predicted enzyme repertoire found in the genome sequence, and the associated network of biochemical reactions with substrates and products. When assuming that enzymatic activity and substrate specificity of orthologs are conserved, the established metabolism in model organisms can serve as a Rosetta Stone for other organisms (Ideker et al., 2001). GEMs allow the integration of miscellaneous omics data and prior knowledge about the metabolic properties of an organism (Zhang and Hua, 2016). Furthermore, they can facilitate *in silico* identification of essential genes, reactions, or metabolites by predicting the effect of enzyme knockouts on the functioning of the system (Chavali et al., 2012). Since metabolism is profoundly connected to all other systems in the cell, a GEM can be used as a proxy to describe the phenotypic state of an organism and serves as a framework to guide future experimental research (McKnight, 2010).

BOX 1 **Genome-scale metabolic models**

Metabolism is a complex system of thousands of biochemical reactions, most of which are catalyzed by metabolic enzymes. GEMs have been developed as an effective way to generate hypotheses about cellular metabolism (Yurkovich and Palsson, 2016). A GEM is based on the repertoire of metabolic enzymes encoded in the genome, which are cross-referenced with biochemical databases or template models to obtain a set of biochemical reactions that can transform substrate metabolites into products. These reactions are interconnected by shared substrates or products, forming a complex network that is typically divided over several subcellular compartments, such as the mitochondria and the cytosol. The metabolic network can be represented by a sparse integer matrix, denoting the stoichiometry of substrate and product metabolites of each reaction.

GEMs are assumed to be in steady state, which means the net uptake of nutrient mass is equal to the net production of biomass, implying there is no net accumulation of metabolites. This simplifies the model to a system of linear equations. Each reaction in the GEM is considered to have a flux, i.e., a steady-state reaction rate. The most popular method to model metabolism in this framework is called flux balance analysis (FBA; Orth et al., 2010). An assumption is that cells have a specific objective, often maximization of growth (production of biomass) or minimal usage of energy (García Sánchez and Torres Sáez, 2014). Linear optimization can find values for all fluxes that attain the specified objective, for instance, maximal flux toward biomass precursors. The production of biomass is modeled as a pseudo-reaction that consumes all biomass precursors (e.g., amino acids for proteins) with appropriate stoichiometry, often manually implemented based on experimental data (Feist and Palsson, 2010). As there is an infinite number of solutions that allow biomass production, constraints need to be implemented to find a single set of fluxes. Thermodynamic constraints can implement an upper and lower bound on each flux, specifying that reactions are either bidirectional (flux can be either negative or positive) or irreversible (Either the upper or lower bound is zero.) These constraints significantly limit the number of possible outcomes of the optimization problem. The flux values that yield a maximal value for the specified objective, within the constraints, are then selected as an optimal solution.

The integration of omics data can be used to impose additional model constraints or objective functions (Bordbar et al., 2014). Since most reactions of a GEM are associated with one or more genes, optimization can take into account gene expression and calculate the fluxes that concur with underlying gene expression. For instance, the INIT algorithm (Agren et al., 2012) maximizes a global score, which increases when “expressed” reactions have a flux and decreases when “non-expressed” reactions have a flux. As a result, the optimal solution depends on the expression of genes (i.e., context-specific submodels), which enables the comparison of fluxes between transcriptome conditions. Similarly, metabolomics data can be used to calculate fluxes that attain the presence of measured metabolites. Note that these are only two specific examples of omics integration into GEMs, in a rapidly expanding set of methods (Lewis et al., 2012).

The linear program of a GEM can often be solved in a matter of seconds on modern computers, making it a powerful computational tool to predict the effect of perturbations to GEMs (Peyraud et al., 2018). For instance, the impact of a different growth medium can be analyzed (i.e., change of nutrient uptake reactions), or genes/reactions can be iteratively removed from the model to investigate the effect on the metabolic fluxes. Reactions that have large effects on the flux distribution or biomass production upon removal suggest biological relevance (Chavali et al., 2012). Essential genes or reactions can be predicted when their elimination yields an infeasible problem, i.e., no solutions respecting the implemented constraints (e.g., no biomass flux possible after removal; Pratapa et al., 2015). Similarly, synthetic lethal gene or reaction pairs can be identified that will only impair biomass production upon simultaneous deletion.
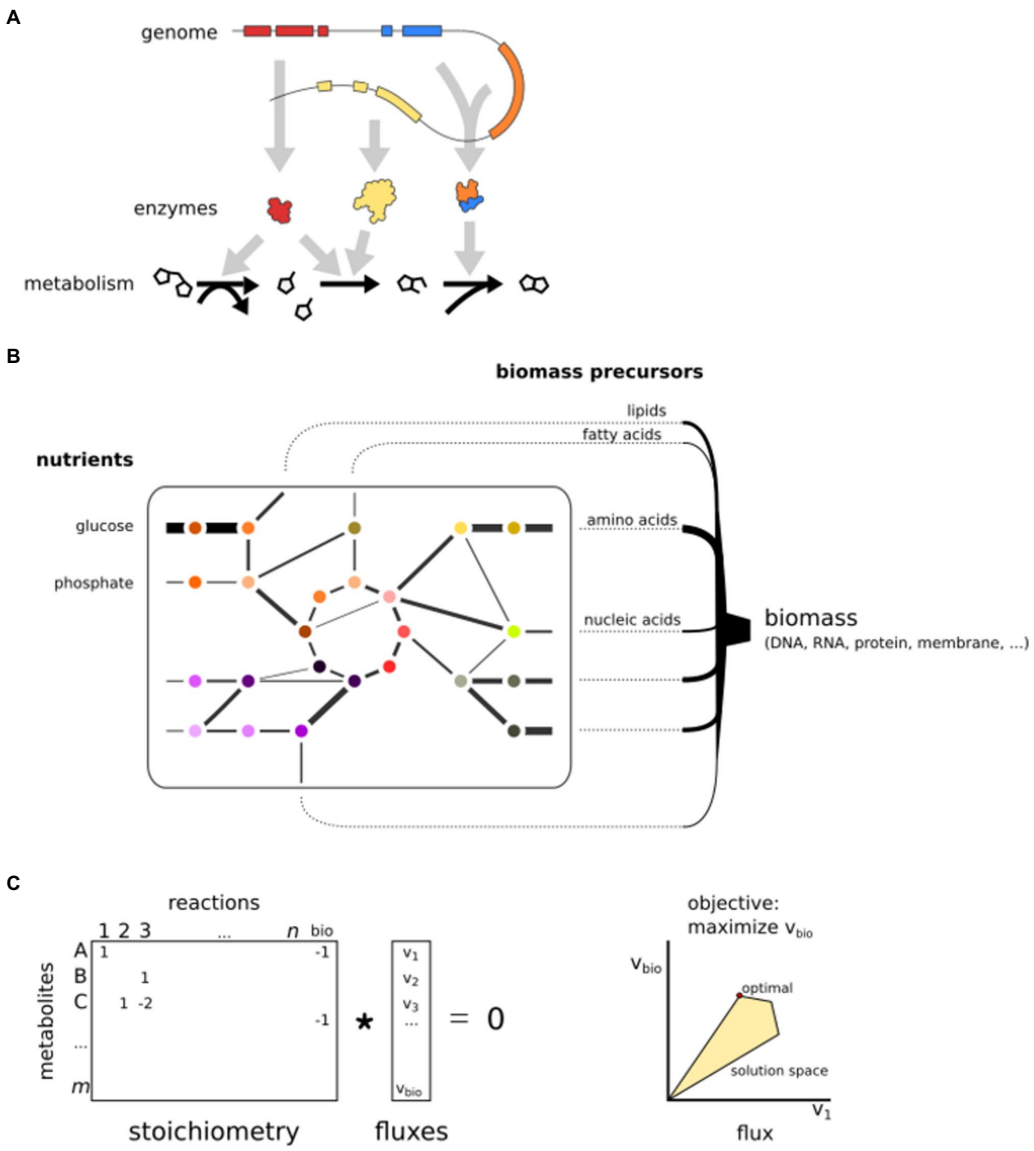

Basic principles of GEMs. **(A)** GEMs are reconstructed from a genome sequence and connect enzymes to reactions. **(B)** A GEM is a network that simulates fluxes from nutrient uptake to the production of biomass precursors. **(C)** The mathematical representation of a GEM. Reactions are stored in a stoichiometric matrix that is multiplied by a vector of fluxes. The solution space is limited by constraints. Here, only two fluxes are shown, but the optimization is in *n* dimensions.

Because of their integrative nature, by relating genes with reactions and metabolites, GEMs can serve as a knowledge base for species-specific information on the biochemical capacity of an organism, as deduced from its genome and from prior knowledge. Prior knowledge typically integrated in a GEM includes, for instance, the nutrients taken up and the metabolites produced in the form of biomass or secondary metabolites. Importantly, prior knowledge should be used to correct the model where automated methods are limited, such as the inference of species-specific enzymatic substrates, gene–protein–reaction associations (i.e., which genes catalyze what reactions, including subunits and isozymes), subcellular localization of enzymes, directionality of reactions, and substrates of transporters (Thiele and Palsson, 2010). Next to a knowledge base, GEMs provide a scaffold for the integration of additional omics data (O’Brien et al., 2015). A cornerstone of systems biology is the continuous integration of new data and new information into existing GEMs, with the aim to improve the quality of a GEM and make it more trustworthy.

The main purpose of GEMs is enabling flux simulations that can be used to investigate system complexity and dynamics (Orth et al., 2010). For instance, this can help unravel which flux distributions are thermodynamically optimal for growth (biomass production)—sometimes in various transcriptomic contexts. Moreover, these simulations can be used to investigate the system’s robustness to induced perturbations (e.g., gene deletions), yielding testable model-driven hypotheses. This process is key for the cyclic process in which model predictions are experimentally validated, driving technological advance, allowing for the integration of new data, and again enabling generation of new hypotheses (Kitano, 2002). A continuous cycle of improvements with additional data and knowledge can eventually lead to a highly predictive model to provide a deeper understanding of the molecular systems of an organism.

## Systems Biology of Pathogens and Host–Pathogen Interactions

Systems biology offers a powerful toolbox to study pathogens and their relation with their hosts (Horn et al., 2012; Durmuş et al., 2015; Dix et al., 2016; Peyraud et al., 2017; Cesur et al., 2018). Pathogens and hosts often interact extensively on all molecular levels, i.e., metabolic, protein, and DNA/RNA (Figure 1). Co-evolution shapes the host’s immune system to be able to recognize the presence or action of a pathogen and to activate immune responses (Cook et al., 2015). To counter these processes and to facilitate infection, pathogens secrete virulence factors in the form of proteins (effectors), small RNAs, or (secondary) metabolites (Frantzeskakis et al., 2019). The symbiosis between pathogen and host can be regarded as a single intertwined system separated into different compartments (Olive and Sassetti, 2016). Thus, the reconstruction of a model for the overall system can help to characterize pathogen–host interactions and their dependencies at unprecedented scale and detail.

Systems biology has been used in the study of various pathogens or pathogen–host interactions to identify drug targets or key factors that allow pathogens to interact with their host (Durmuş et al., 2015). In pathogen–host interactions, protein–protein or small RNA interaction networks have been investigated using graph theory to identify pathogen effectors and their host interactors, in which network centrality or degree is considered a proxy for functional importance (Durmuş Tekir and Ülgen, 2012). GEMs can simulate the system-wide metabolic fluxes of a pathogen and help identify important genes, reactions, and metabolites, which can inspire novel control strategies (Chavali et al., 2012). Not surprisingly, the first GEM ever generated was for a microbial pathogen, i.e., the bacterium *Haemophilus influenzae* that causes disease in humans (Edwards and Palsson, 1999). Since then, GEMs have been reconstructed for many more pathogens, such as the tuberculosis bacterium *Mycobacterium tuberculosis* (Kavvas et al., 2018; Rienksma et al., 2018) and the human and animal parasites of the genera *Plasmodium* (Plata et al., 2010; Stanway et al., 2019) and *Leishmania* (Subramanian et al., 2015; Sharma et al., 2017; Chauhan and Singh, 2019). Moreover, some GEMs integrated pathogen and host, thereby providing insight into the metabolic fluxes throughout infection (Bordbar et al., 2010; Huthmacher et al., 2010; Bazzani et al., 2012).

In contrast to human pathogens, plant pathogens have thus far hardly been studied with a systems biology approach (Peyraud et al., 2017). Similar to human pathogens, plant pathogens have a major negative impact on the well-being of their hosts. Plants are crucial for generating the oxygen (O_2_) we breathe, for sequestering CO_2_ and maintaining the balance in the global ecosystem, and for the production of food and feed. However, plants are under constant threat of pathogens, such as fungi, oomycetes, bacteria, and viruses. In agriculture, the resulting yield losses can be substantial, reaching up to 30% (Savary et al., 2019). To combat plant diseases, a better understanding of plant–pathogen interactions is required. Thus far, there are, however, only few examples where systems biology was applied to provide insight into the molecular mechanisms underlying plant–pathogen interactions. In one study that was based on yeast-two-hybrid screenings, a protein–protein interaction network of *Arabidopsis thaliana* and pathogens of three kingdoms uncovered that effectors from different pathogens convergently target the same host proteins (Weßling et al., 2014). In a more recent study based on mass spectrometry analyses of immunoprecipitated effector–host target protein complexes in *Nicotiana benthamiana*, the deduced protein–protein interaction network revealed the cellular vesicle trafficking machinery as a major effector-targeted process (Petre et al., 2021). In other studies, GEMs have been reconstructed for the bacterial plant pathogens *Ralstonia solanacearum*, *Xanthomonas oryzae*, and *Pectobacterium parmentieri* (Peyraud et al., 2016; Zoledowska et al., 2018; Koduru et al., 2020), and for the fungus *Sclerotinia sclerotiorum* (Peyraud et al., 2019). However, despite the abundance of omics data for many plant pathogens, very few have been analyzed from a systems biology perspective.

## Oomycete Pathogens

Oomycetes are filamentous eukaryotes that resemble fungi in terms of morphology but evolved independently from fungi (McGowan and Fitzpatrick, 2020). In the tree of life, oomycetes are clustered with the brown algae and diatoms in the Stramenopile lineage (Beakes et al., 2011; Keeling and Burki, 2019). Many oomycetes are plant pathogens, while others are animal pathogens, parasitize on other microbes, or live as saprophytes (Derevnina et al., 2016b). The plant pathogenic oomycetes vary in lifestyle, including necrotrophs that swiftly kill their hosts and feed off dead plant material (Fawke et al., 2015) and biotrophs that need living host tissue to infect, feed, and proliferate. Most of the biotrophic oomycetes, such as the white rusts and downy mildews, are obligate pathogens implying that they cannot grow outside a living host (Baxter et al., 2010). They usually specialize on just one plant species and hence have a very narrow host range. Others are known as hemibiotrophs; they live as biotrophs during the initial phase of the disease cycle but switch to a necrotrophic lifestyle later on. *Phytophthora* species are mostly hemibiotrophs. To date, over 150 *Phytophthora* species have been described, all with their own specific host range, sometimes limited to one or few plant species within one family but more often multiple plant species from different families. Well-known narrow host range species are *Phytophthora infestans* that causes late blight disease on potato and tomato, and *Phytophthora sojae*, the soybean root and stem rot pathogen. Examples of broad host range species are *Phytophthora ramorum*, the sudden oak death pathogen, *Phytophthora capsici* that causes stem and fruit rot on many vegetables, and *Phytophthora palmivora*, a devastating pathogen on tropical crops such as cacao and date palm (Kamoun et al., 2014; Perrine-Walker, 2020). The type species of the genus is *P. infestans* which caused the Irish potato famine in the mid-nineteenth century. It was initially named *Botrytis infestans* but later renamed by de Bary (1876) who provided the formal proof that this organism is the causal agent of potato late blight and coined the term *Phytophthora*, Greek for plant (phyton) and destruction (phthora). Its arrival in Europe marked the birth of plant pathology as a discipline and ever since *P. infestans* has been a favorite subject of investigation (Turner, 2005). Technological advancements in molecular genetics and genomics over the last four decades further boosted the interest in studying this important plant pathogen (Turner, 2008). Although still considered a model organism for oomycetes, in research it is losing ground to other species that have smaller genome sizes and are more amenable to genetic modification.

## The Interplay Between Host and Oomycete Pathogens

Biotrophic plant pathogens typically adhere to leaves or roots, break physical barriers, and scavenge nutrients from their host, while suppressing the host’s immune system (McDowell, 2011). They achieve this by depositing a large variety of enzymes and effector proteins in the apoplast or inside the plant cell that help in paving the way for a successful infection (Whisson et al., 2016). During infection, these biotrophs grow as filamentous hyphae inside their hosts. They colonize the apoplastic space in the leaf mesophyll and form feeding structures, so-called haustoria, inside host cells. At the site of the haustoria and the apoplastic hyphae, pathogen and host form a close interface through which effectors, enzymes, and small molecules can be exchanged. This interplay often involves a prolonged symbiosis in which the pathogen feeds off the plant for growth and reproduction (Judelson and Ah-Fong, 2018).

The ability of oomycetes to live in close symbiosis with a host drives continuous adaptations of both pathogen and host. Oomycetes have dynamic genomes that allow swift adaptation (Leesutthiphonchai et al., 2018). These genomes typically harbor hundreds of effector genes (McGowan and Fitzpatrick, 2017) Comparative genomics has revealed that obligate biotrophic pathogens have suffered extensive gene loss as a result of their biotrophic lifestyle (Kemen and Jones, 2012; Fletcher et al., 2018). This adaptive capacity facilitates the evolutionary “arms race” between oomycete effectors and host resistance genes (Wang et al., 2019b), but also allows adaptation of the core cellular machinery of pathogens, including metabolism and signal transduction, leading to various unique properties (Judelson, 2017). For instance, oomycetes have several genes encoding unique proteins with novel domain combinations (Seidl et al., 2010; van den Hoogen and Govers, 2018a), as well as a number of horizontally transferred genes coding for proteins with functions in metabolism (Richards et al., 2011). Oomycetes are osmotrophs, which means they secrete enzymes to digest large molecules (polymers) extracellularly and import the resulting small molecules as nutrients (Richards and Talbot, 2013). This process is facilitated by a broad array of transporter proteins, suggesting that a plethora of host compounds can be taken up during infection (Abrahamian et al., 2016). However, some nutrients are indispensable for oomycetes. For instance, several oomycetes lost the ability to synthesize sterols (Wang et al., 2021). These sterol auxotrophs secrete elicitins, oomycete-specific proteins that are thought to be sterol carriers and likely exploited for recruiting sterols from the environment (Derevnina et al., 2016a). Moreover, most oomycetes are auxotrophic for thiamine, a vitamin that acts as a cofactor in carbohydrate catabolism (Hohl, 1991). Culturable oomycetes can be grown *in vitro* and seem to prefer amino acids as a substrate (Hodgson, 1958; Ah-Fong et al., 2017b), but can utilize a wide variety of substances. Due to the complex nature of cellular metabolism, it is currently unclear which nutrients are more important than others, and how the differential usage of nutrients might influence infection. It is also not so easy to readily gain such knowledge. Many oomycetes are hard to culture and require complex media for *in vitro* growth, often prepared from seeds such as rye kernels, peas, or lima beans. This obviously complicates biochemical assays to investigate their metabolism, for which knowledge of the precise growth substrates is mandatory. For obligate biotrophs that exclusively grow inside their living host, it is even more challenging; unravelling the precise composition of their diets is extremely difficult if not impossible (McDowell, 2011).

Validation of the role of enzymes or transporters in metabolism by targeted mutagenesis is another challenge when investigating oomycetes. For several *Phytophthora* and *Pythium* species, successful DNA transformation has been described but transformation efficiencies are often relatively low. Until recently, functional gene analyses relied on gene silencing or overexpression of the target gene with the disadvantage that the variability in silencing or overexpression levels and potential off-target effects make phenotypic characterization of the transformants complicated and labor-intensive. A major breakthrough was the achievement by Fang and Tyler (2015) who published the first successful application of CRISPR-Cas9-mediated gene editing in an oomycete, namely *P. sojae*, and by now, this is a standard method to create gene knockouts in several *Phytophthora* species (Wang et al., 2019a; Pettongkhao et al., 2020). In *P. infestans*, however, CRISPR-Cas9-mediated gene editing was not successful (van den Hoogen and Govers, 2018b). A recent study by Ah-Fong et al. (2021) showed that Cas9 is toxic for this species, but with Cas12a as nuclease they obtained transformants that are viable and have small deletions in the target gene, the *inf1* elicitin gene. This is another major leap forward as it demonstrates the successful implementation of a promising gene editing tool in *P. infestans*.

## Systems Biology on Oomycetes and Oomycete–Host Interactions

Oomycete pathogens are very challenging to control due to their high capacity for adaptation. It is therefore important to reconstruct holistic models that provide mechanistic insight into the molecular systems that allow oomycetes to proliferate and infect their hosts. Ultimately, a system-wide understanding of oomycete–host interactions might provide novel leads for control (Dunphy and Papin, 2018).

Shortly after the first genome sequences of oomycetes were published (Tyler et al., 2006; Haas et al., 2009), it was already proposed to reconstruct predictive models with the aim to reveal mechanisms of oomycete–host interactions (Pinzón et al., 2009; 2010). One of the first studies that reconstructed a partial metabolic network for *Phytophthora* used the network to provide context for predicting horizontal and endosymbiotic gene transfer (Whitaker et al., 2009). In subsequent studies, Seidl et al. (2013) predicted a functional association network in *P. infestans* by projecting genomic, transcriptomic, and comparative genomic data on protein–protein interaction networks of model organisms, while Mukhtar et al. (2011), Weßling et al. (2014), and Petre et al. (2021) created holistic networks uncovering a plethora of interactions between oomycete effectors and potential host targets.

Investigation of a static metabolic network can already provide new biological insights that do not come to the foreground when only smaller subsets of the data are considered. An example is a recent study in which we identified and compared the metabolic enzymes of a broad range of oomycetes with different lifestyles and host preference and investigated their metabolic networks from an evolutionary perspective (Rodenburg et al., 2020). Similar to Thines et al. (2020), we observed lineage-specific pathway loss, and convergent loss of metabolic enzymes in obligate biotrophs reflecting their reduced metabolic capacity and greater host dependency. Intriguingly, the gene losses predominantly affected the periphery of the metabolic network, an insight that remains hidden when solely comparing genomes.

## Modeling *Phytophthora Infestans* Metabolism to Predict Pathogen–Host Interactions

In 2018, we (Rodenburg et al., 2017) and Botero et al. (2018a) presented the first GEMs of P. infestans. Despite the slightly different reconstruction and analysis approaches and the more extensive transcriptome data set in Botero et al. (2018a) including *in planta* life stages, the overall findings were comparable. For instance, the models both pinpointed fatty acid biosynthesis as a key process in oomycetes and in both models, condition-specific metabolic patterns were apparent.

As input for our modeling (Rodenburg et al., 2017), we extracted information on *P. infestans* metabolism from the literature and identified all putative enzymes encoded in its genome by homology-based enzyme annotation. We then divided reactions over the subcellular compartments and inspected the model topology to gain insight into the biochemical processes in each compartment. A further refinement was the integration of transcriptome data; the resulting life stage-specific models showed a sharp contrast in metabolic activity between sporangia and hyphae. In oomycetes, the sporangia, which are asexual spores, likely rely on stored nutrient reserves, such as glucans and fatty acids, that are catabolized for energy production (Judelson, 2017). When sporangia disperse and reach a suitable plant surface, zoospores are released and encyst. The cysts then germinate and form an appressorium-like structure at the tip of the germ tube.

For reconstruction of a GEM, information on growth phenotypes on defined substrates is pivotal and the availability of knockout mutants would greatly contribute to validate the predictions (Nakahigashi et al., 2009; Monk et al., 2014). Unfortunately, experimental data on metabolism in *P. infestans* are very limited and, as described above, tools for knockout mutagenesis still have to be further optimized. The information that is available in the literature includes data on minimal *in vitro* growth substrates (Hohl, 1991), verified subcellular localizations of enzymes (López-Calcagno et al., 2009; Abrahamian et al., 2017), and capacity to produce a mixture of long-chain polyunsaturated fatty acids (Griffiths et al., 2003; Sun et al., 2012). Perhaps the most important limitation that we faced when reconstructing the *P. infestans* GEM was the lack of knowledge on biomass composition, i.e., the stoichiometry of *P. infestans* biomass precursors (Feist and Palsson, 2010). The biomass composition relates the fluxes in the model to a hypothetical growth rate, and as such, it can be used as a proxy for metabolic fitness. Because a precise description of *P. infestans* biomass composition was not available, we estimated it from the literature but ignored relative abundance (stoichiometry; Rodenburg et al., 2017). This rendered quantitative flux predictions infeasible, but still allowed us to investigate the model for connectivity and importance of different nutrients (Rodenburg et al., 2019). Similar challenges were faced by others modeling pathogens. Tymoshenko et al. (2015), who published a GEM for the human parasite *Toxoplasma gondii*, also reconstructed a biomass composition from the literature, ignoring stoichiometry. For a GEM of *Leishmania donovani*, Sharma et al. (2017) chose to infer the biomass composition from a *Plasmodium* GEM. For oomycetes, it may be an option to adopt the biomass composition from curated GEMs of closely related organisms, such as the brown algae *Phaeodactylum tricornutum* (Levering et al., 2016) or *Ectocarpus siliculosus* (Prigent et al., 2014), but this should be weighted against the risk of introducing new biases and uncertainties. After all, the similarity of biomass composition between brown algae and oomycetes is unknown and, further complicating matters, the biomass composition of *P. infestans* in different life stages appears to be radically different (Grenville-Briggs et al., 2008).

**Figure 2:**
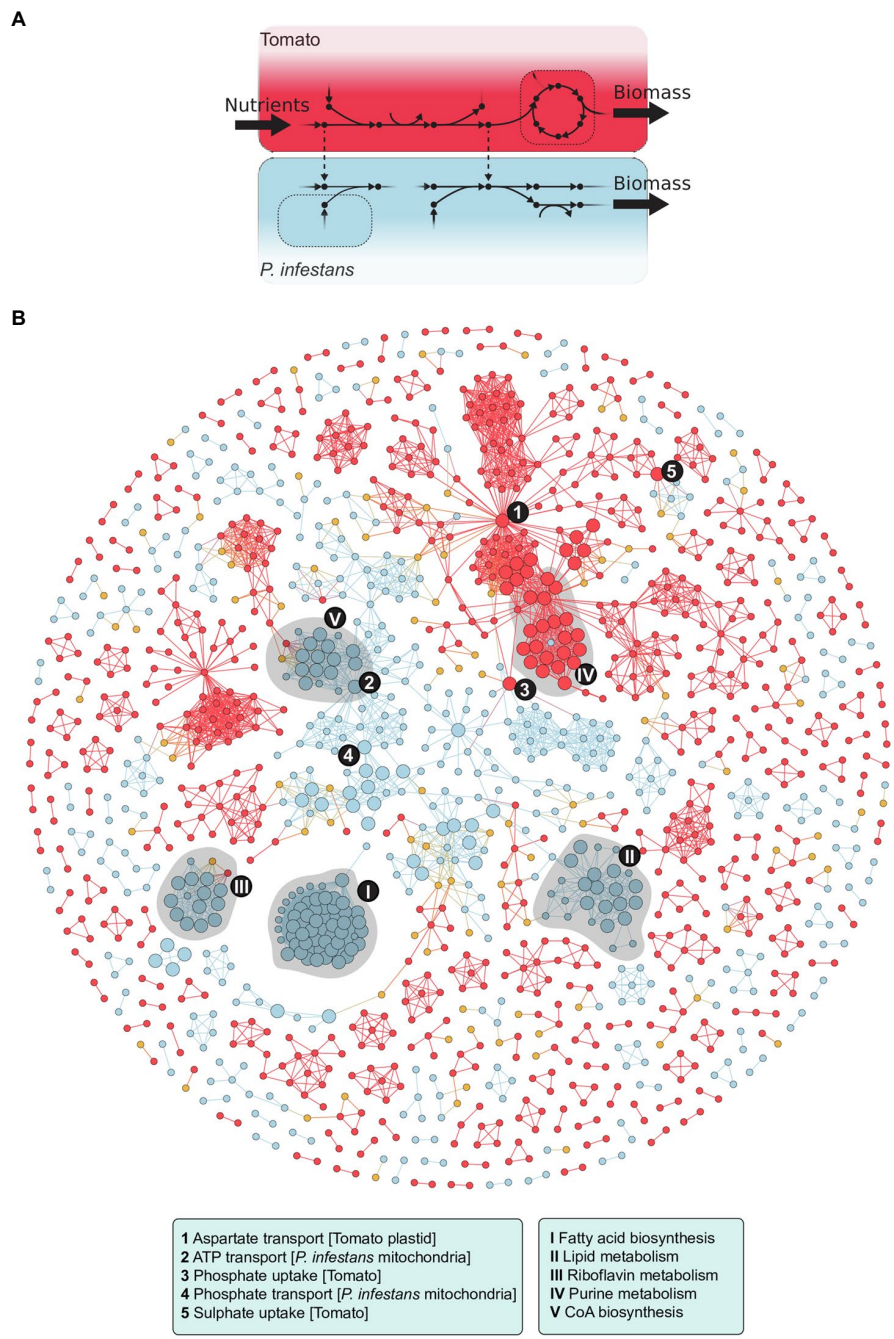
The integrated *P. infestans*–tomato model (reproduced and adapted from Rodenburg et al., 2019). **(A)** Diagram in which dots represent metabolites, arrows reactions, and dotted lines host–pathogen transport reactions. **(B)** Flux coupling analyses of the model identified 77 coupled (i.e., topographically related) *P. infestans*–tomato reaction pairs which are shown in the graph. Nodes represent reactions in tomato (red) or *P. infestans* (blue) and host–pathogen transport (yellow). Edges represent coupling between those reactions. The nodes with the largest diameter represent 112 reactions in *P. infestans* and 35 in tomato that were found to be essential for *P. infestans* biomass production. The various processes listed in the boxes are represented by highly connected nodes (1 to 5) and in shaded clusters (I to V). Further details in Rodenburg et al. (2019).

Although the GEM of a plant pathogen is in principle suitable to predict essential metabolic genes and reactions, and to simulate growth (biomass production) *in vitro*, it is less informative for predicting the pathogen’s metabolism during *in planta* growth. We addressed this by integrating our initial *P. infestans* GEM (Rodenburg et al., 2017) with a tomato GEM published by Yuan et al. (2016), resulting in a multi-compartment metabolic model of the *P. infestans*–tomato interaction (Figure 2A; Rodenburg et al., 2019). We used modeling techniques such as flux coupling analysis, to identify metabolic reactions in tomato that are of importance to fluxes in *P. infestans* metabolism. An example is thiamine biosynthesis that is required to supply the thiamine auxotrophic pathogen with this essential vitamin (Rodenburg et al., 2017). In this way, we build a GEM of a pathosystem by approaching it as a single system and demonstrated that this GEM can be used to predict metabolic changes in both host and pathogen (Figure 2B). Botero et al. (2018b) used an alternative approach: They constructed a GEM for potato (*Solanum tuberosum*) and modeled the metabolic changes in the plant when challenged by *P. infestans*, based on transcriptome data.

Nutrient uptake by a pathogen depends on location and stage of infection; however, a GEM of a pathosystem typically lacks resolution to take these factors into account. The first close encounter between a *Phytophthora* pathogen and a plant is when a germ tube emerging from a sporangium or cyst senses a suitable surface and starts swelling at the tip. Host entry by *Phytophthora* is facilitated by a mechanical slicing mechanism that breaches the epidermal cells (Bronkhorst et al., 2021). Haustoria emerge from hyphae that colonize the apoplast and enter the mesophyll cells (Judelson, 2017). It is often assumed that these haustoria are the main site of nutrient uptake, as is the case for various plant pathogenic fungi (Wang et al., 2018). However, many oomycetes do not form haustoria (Fawke et al., 2015), and haustoria make up only a very small proportion of the total hyphal biomass (~2%), raising the question whether haustoria are truly the main site of nutrient uptake (Judelson and Ah-Fong, 2018). The plant apoplast is a nutrient-rich environment and might be the main site of nutrient uptake (Chen, 2013). Haustoria are nonetheless very important for the host–pathogen interaction. They form the site from where the pathogen deposits so-called cytoplasmic effectors into the plant cell for suppression of immune responses (Boevink et al., 2020). The host recognizes the intracellular host–pathogen interface created by the haustorium as the site where defense responses have to be activated and, for example, relocate the nucleus to the interface (Wang et al., 2017). Next to cytoplasmic effectors, the pathogen secretes apoplastic effectors, so also in the apoplast host and pathogen interact and likely this involves exchange of signals and compounds.

Because of the specialized tasks of the haustorium and hyphae in the apoplast, hyphal cells likely have a “division of labor,” which implies that the biological processes are tailored for the specific region of infection. This phenomenon was recently modeled for the fungal plant pathogen *Sclerotium sclerotiorum*, by mapping the transcriptome of the apex and the center of infection to a multi-cell GEM (Peyraud et al., 2019). In the reconstruction of the integrated *P. infestans–*tomato GEM, we did not explicitly discriminate between the different sites of infection but rather focused on changes over time (Rodenburg et al., 2019). We integrated dual-transcriptome data obtained from infected tomato leaves. A time course of a full infection cycle was sampled with intervals of 4h during 4days (2–6days after inoculation), resulting in 25 submodels. This revealed various switches in metabolism and differential nutrient usage over time, with a “division of labor” of the two partners. As infection progresses, *P. infestans* performs less *de novo* synthesis of metabolites and scavenges more metabolites from tomato. This example nicely demonstrates how one can analyze transcriptome data in a system-wide context. In concordance with related transcriptome studies (Abrahamian et al., 2016; Ah-Fong et al., 2017a), the transcriptome-based submodels reflected reduced metabolic activity in the sporangial stages of *P. infestans*, and nutritional changes in the transition from a biotrophic to a necrotrophic stage of infection on tomato leaves (Rodenburg et al., 2017, 2019). Importantly, because these transcriptomic changes were analyzed in the context of a GEM, results were subject to the imposed model constraints (steady-state, reaction thermodynamics) and thereby to the topology of the metabolic network (Hyduke et al., 2013). The transcriptomic changes are interpreted in terms of ensuing differences of metabolic fluxes, and as such, this system-wide approach can be more informative than the differential expression analyses of individual genes. The integrated metabolic model provides a framework to simulate the metabolic fluxes occurring during infection and as a result, new insights in the kind of nutrients that *P. infestans* extracts from its host during the subsequent phases of the infection cycle.

Metabolic enzymes are located in various organelles, and hence, specific metabolic processes take place in different parts of the cell. In the reconstructed *P. infestans* models, this compartmentalization was taken into account to represent the spatial distribution of metabolic pathways in different subcellular compartments (Rodenburg et al., 2017, 2019). The transporters and channels responsible for transfer of metabolic substrates across membranes were modeled by integrating transport reactions (Thiele and Palsson, 2010). Transporters typically have a wide substrate range. Because of the difficulty of predicting the substrate based on protein sequence, transporters are often manually added to a GEM based on prior knowledge. This is particularly challenging when creating an integrated pathogen–host metabolic model, considering that metabolite transport across membranes is pivotal to pathogen nutrition. In modeling the *P. infestans–*tomato interaction, we chose to not manually add transport reactions (Rodenburg et al., 2019). Because too little is known about *P. infestans* nutrition *in planta*, manually adding host–pathogen transport reactions would bias fluxes toward a predefined set of nutrient transporters. Since one of our goals was to predict the nutrient pool of *P. infestans* during tomato infection, we chose to draw conclusions based on the optimal fluxes in the model. In other words, the transport reactions in our models were largely based on network topology. Depending on the objective function, the most optimal set of transporters had a nonzero flux. The downside of this approach was that we could not consider bidirectional transport, as this would imply unrestricted metabolite exchange between host and pathogen. This could lead to scenarios where the host would utilize certain *P. infestans* metabolites for profit, which, from a biological point of view, is not plausible. In reality, however, metabolite exchange is likely a two-way process, with the host providing nutrients and the pathogen secreting metabolites, for example, as waste products or virulence factors. There is a clear knowledge gap on the metabolic exchanges that *P. infestans* maintains with its environment. To fill this gap, broad substrate screening could be performed using various growth media and different time points of mycelial growth, combined with comparative metabolomics using mass spectrometry, guided by GEM predictions.

## High-Quality Genome Data are Essential for Reconstructing Reliable Genome-Scale Metabolic Models

GEMs are reconstructions of cellular systems that can be used to investigate the genotype–phenotype relationship: How do the genes encoded by the genome result in the complex biological system that we observe (Yurkovich and Palsson, 2016)? Quality and insights derived from genome-scale models therefore critically depend on the quality of the genome sequence and gene annotation.

**Figure 3:**
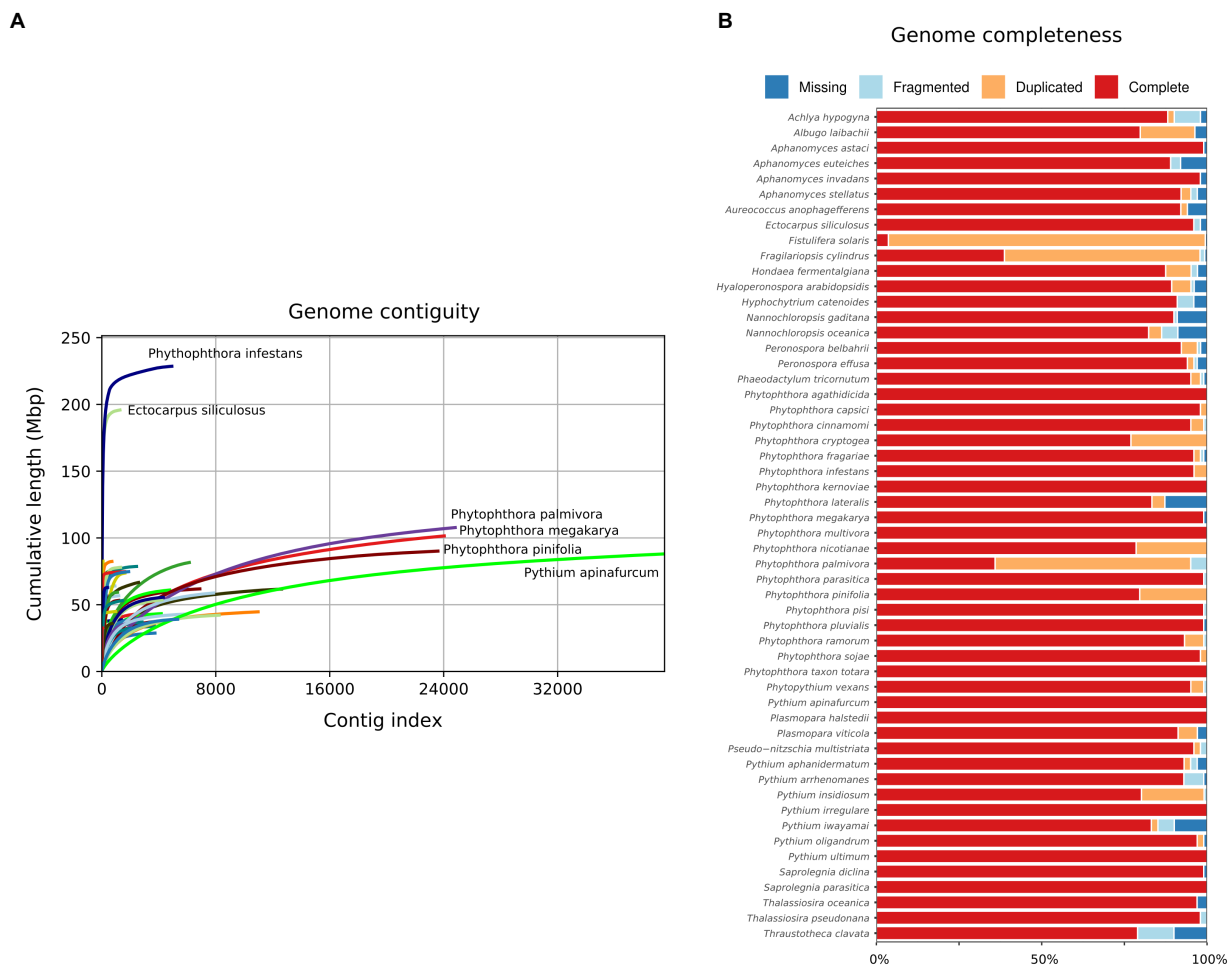
Statistics for 54 published Stramenopile genome sequences including 42 oomycetes. **(A)** Genome accumulation curves derived from Quast (Gurevich et al., 2013), showing cumulative genome size when contigs are ordered from large to small. X-axis represents the number of contigs, and Y-axis represents genome size. **(B)** Presence of near-universal single copy orthologs in the genomes, as determined by BUSCO. Species are in alphabetical order. Colors indicate the completeness of the detected BUSCO genes. For further details on the genome sequences used for these analyses see Rodenburg et al. (2020).

Obtaining a high-quality genome assembly is still challenging, in particular for the more complex eukaryotic genomes that often have a high abundance of repetitive elements and are typically diploid, or sometimes even polyploid or aneuploid (Nagarajan and Pop, 2013). Sequencing and comparative analyses of the first oomycete genomes in 2006 (*P. sojae* and *P. ramorum*) and 2009 (*P. infestans*) revealed that these species profoundly differ in genome size and content (Tyler et al., 2006; Haas et al., 2009). *P. infestans* has a large genome compared to its close relatives, primarily due to the high abundance of transposable elements, constituting roughly 74% of its genome. Oomycete genome assemblies are often still rather fragmented (Figure 3A; McGowan et al., 2018), in particular compared to fungal plant pathogens for which genome assemblies are nowadays often near-complete (Faino et al., 2015). Only over the last few years, near-complete genome assemblies have been published for some oomycetes (Fletcher et al., 2019; Malar et al., 2019; Stajich et al., 2021). Despite the fragmented genome assemblies, almost all oomycete genome sequences comprise over 95% of the near-universal single-copy conserved orthologs, as determined by BUSCO (Figure 3B; Seppey et al., 2019), suggesting that a significant proportion of the coding genome is captured.

Another major challenge is to correctly identify the open reading frames with associated exon boundaries (gene models) within the assembled genome sequences (Salzberg, 2019), which is even more problematic in fragmented genome assemblies (Denton et al., 2014). Eukaryotic genomes are typically annotated using gene predictors trained on the parameters of high-quality gene models from closely related species and aligned transcriptome data (Yandell and Ence, 2012). Annotation of the first two sequenced *Phytophthora* genomes was performed using a gene predictor trained on expressed sequence tags (Tyler et al., 2006). Many subsequent oomycete genome annotations were performed by gene predictors trained on the gene models in other oomycete genomes (McGowan and Fitzpatrick, 2017). However, the error rate in predicted gene models is still high, emphasizing the need for specifically searching for potentially missing gene models when mining genome sequences of oomycetes. Consequently, the field would benefit greatly from manual, and preferably community-driven, curation efforts of genome annotations (Rödelsperger et al., 2019). This would be valuable even when applied to only a single species, preferably one with a near-complete genome assembly, which can then be used as a template to re-evaluate gene models of other oomycetes and train gene predictors.

Reliable functional annotation of the predicted proteome is an additional prerequisite for identifying the fundamental components of genome-scale models. When performing an automated proteome annotation for 54 Stramenopile species, we found that the majority of predicted proteins could not be assigned to a KEGG orthologous group (KO), a system to cluster protein orthologs with validated functions (Mao et al., 2005), and this hampered the association of these proteins with a putative function (Rodenburg et al., 2020). Many effector genes in oomycetes (e.g., those encoding RxLR effectors) lack functional associations (McGowan and Fitzpatrick, 2017). One should consider that orthologs in distantly related species are inherently harder to detect than in closely related ones, which poses a problem in homology-based annotations. However, even in the best studied eukaryotic model organism, the yeast *Saccharomyces cerevisiae*, about 20% of all genes lack any functional association (Wood et al., 2019). Lineages that are evolutionarily distant to model organisms—such as the oomycetes—have an even less functionally characterized proteome, partly because of limitations of homology-based inference of protein functions, but most importantly, because of lack of experimental characterization. An effective and sensitive tool to predict protein functions by homology is offered by hidden Markov models (HMMs). HMMs are trained on a multiple sequence alignment of a predefined cluster of homologous protein sequences and weigh conserved sequence regions heavier than variable regions. Therefore, HMMs are particularly suitable for detecting protein domains, as these are often highly conserved to retain their biological function (Pearson, 2013). KOs are predefined ortholog clusters and powerful resources to train HMMs (Aramaki et al., 2019). In our studies, we used KO-based HMMs to identify orthologs of metabolic enzymes in oomycetes and their close relatives.

In omics-based bioinformatics studies, it is common practice to search for overrepresentation (enrichment) of functional annotations in differentially abundant molecules such as mRNA or proteins (Reed et al., 2006; Bordbar et al., 2014). Over the last decade, there has been a continuous flow of studies presenting oomycete comparative genomics, transcriptomics, proteomics, proteogenomics, and metabolomics data (Andronis et al., 2020; McGowan and Fitzpatrick, 2020). These large-scale omics datasets are analyzed to understand how oomycetes evolve, reproduce, and interact with their hosts. Despite omics studies being indispensable to investigate the transcriptional and/or translational responses of both pathogen and host during infection, these studies are often biased, as usually only a subset of functions is investigated and these are not necessarily representative or causative for the complex phenotype. Moreover, proteomic and metabolomic samples typically only capture the most ubiquitous molecules, and as the differential abundance of any molecule may be influenced by subtle changes in the environmental or experimental conditions, the biological implications remain speculative.

## Toward High-Quality Gems of Pathogens and Pathogen–Host Interactions

Systems biology has been recognized years ago as a promising method to study plant pathogens (Pinzón et al., 2009; Pritchard and Birch, 2011). In the last few years, the potato and tomato late blight (*P. infestans*) pathosystem was subject of several systems biology studies (Seidl et al., 2013; Rodenburg et al., 2017, 2019; Botero et al., 2018a,b; Castro et al., 2019; Thines et al., 2020). Nevertheless, systems biology of this pathosystem and many others is still in its infancy. The level of knowledge on the organism to be modeled is key for the success of a systems biology approach. Thus, to arrive at highly predictive models for *P. infestans* or any other pathogen in the near future, *in vitro* experiments need to be performed to gain basal knowledge. In the case of *P. infestans*, valuable information would be, for instance, the substrates that *P. infestans* can assimilate from its environment, as well as its biomass composition and how this changes throughout its lifecycle. There is a lot we can learn from the more advanced metabolic research in other pathosystems. For example, there are now several GEMs for *Plasmodium* spp., some of which are also integrated with GEMs of the host, the red blood cell (Huthmacher et al., 2010; Plata et al., 2010; Abdel-Haleem et al., 2018). The foundation of these GEMs was provided by pathogen–host metabolomics analyses identifying growth substrates (Olszewski et al., 2009). These data were integrated with new omics data and novel biochemical knowledge into GEMs (Bazzani et al., 2012; Carey et al., 2017; Stanway et al., 2019). Interestingly, these models have pinpointed several essential reactions, some of which turned out to be leads for promising drug targets (O’Hara et al., 2014). For protozoan parasites, isotope-labeled growth experiments have been successful to dissect their metabolism during parasitic growth (Kloehn et al., 2016), and it can be anticipated that similar analyses will provide intriguing novel avenues to control oomycete and fungal plant pathogens.

**Figure 4:**
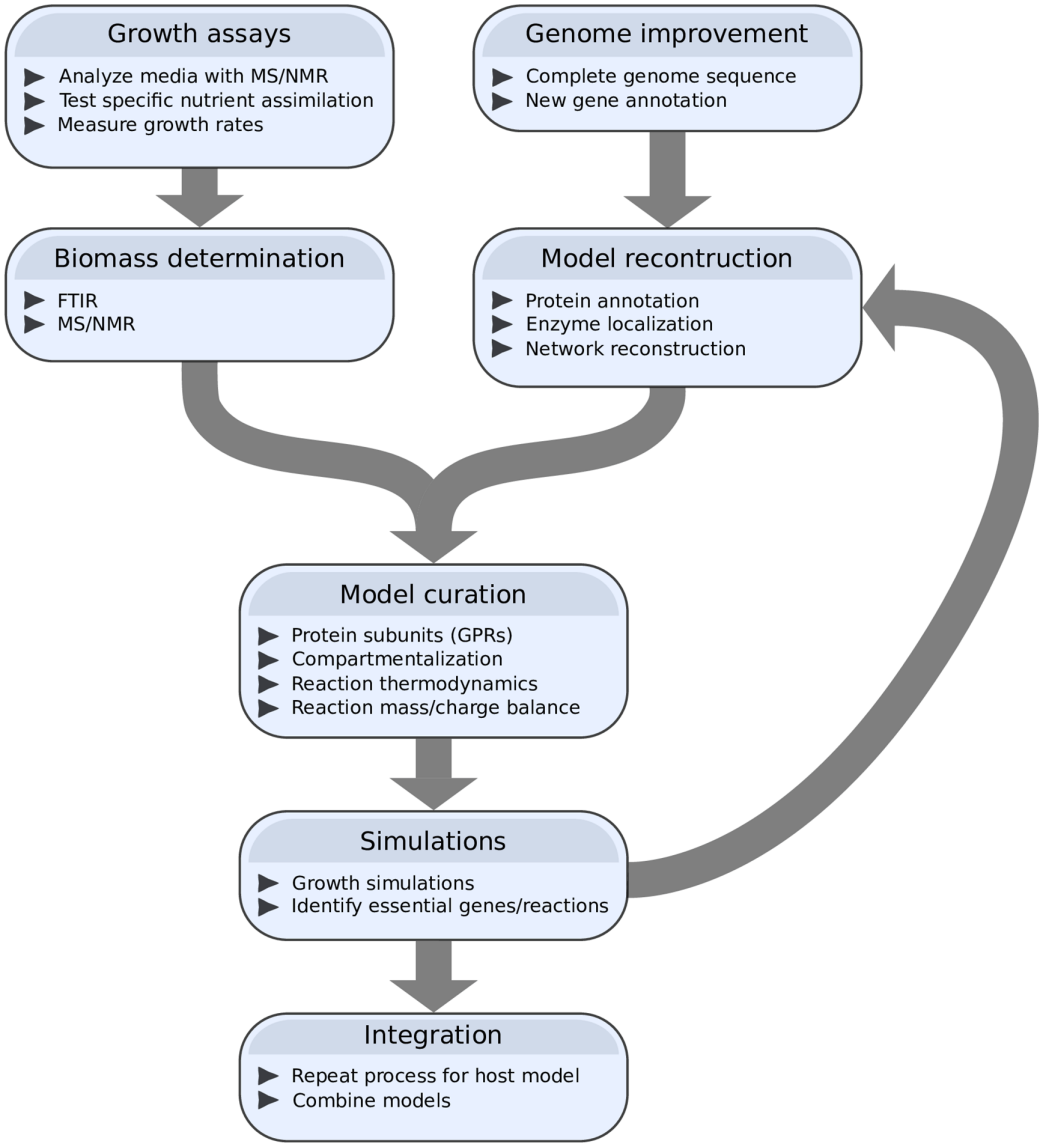
A proposed workflow (including possible methods and analyses) for reconstructing a high-quality genome-scale metabolic model (GEM) for *P. infestans* and, potentially, an integrated GEM for a *P. infestans*/host interaction. Model reconstruction is an iterative process of simulation and model improvement.

To be able to take full advantage of systems biology, several steps to create a higher-quality GEM for *P. infestans* as well as other plant pathogens are needed in the future (Thiele and Palsson, 2010). Obviously, complete functional characterizations of the substrates and characteristics of each individual metabolic enzyme in the pathogen as well as in the host would be ideal, but this seems infeasible in the near future. Nevertheless, significant achievements could be gained from *in silico* and *in vitro* procedures, designed specifically for the purpose of building a high-quality GEM (Figure 4).

First (near-)complete and ideally gapless genome assemblies of pathogens as well as their hosts are required. The genomes need to be sequenced and assembled using novel technologies and advanced assembly methods to attain the complete coding information (Thomma et al., 2016). Gene prediction should be guided by RNA sequencing, homology-based evidence, and metabolics data, and predicted protein sequences should be functionally annotated. Moreover, manual gene model curation should be performed to accurately predict the enzymes and all this information should be linked to knowledge on the biochemical capacity of the pathogen (Fernandes et al., 2019). Such manual curation is time-and labor-intensive, but will ultimately lead to a complete description of the enzyme catalogue encoded in the genomes and thus to better models.

Second, the identified enzymes are then used as input for reconstructing a draft metabolic network. Functional annotation of (subunits of) metabolic enzymes and the associated reactions and metabolites should be curated according to the literature and according to the established protocol (Thiele and Palsson, 2010), pinpointing gene–protein–reaction associations. The metabolic network needs to be manually inspected to identify potentially missing enzymes (gaps), by comparing the reconstructed pathway to reference pathways of well-characterized model organisms, and the annotations need to be revisited to account for apparently missing enzymes (i.e., wrong or erroneous gene annotation). Additionally, these models should be compartmentalized, i.e., reactions should be assigned to the correct cellular location with transport reactions to simulate the transmembrane metabolite fluxes. We and others previously considered at least the cytosol, extracellular space, and the mitochondria essential, since these are hotspots of metabolism. Intracellular transport reactions need to be manually included based on common biochemical knowledge, such as experimental data, textbooks, or literature. We found that specifically for oomycetes the experimental data on transporter substrates are very limited, and it is unlikely that many more transporters are to be characterized, given the labor-intensive process (Savory et al., 2018). Therefore, transport reactions may be inferred from GEMs of related species and by analyzing network topology.

Third, *in vitro* assays can be performed to characterize the pathogen’s growth behavior under different conditions. The ideal medium is similar in composition to the pathogen’s natural hosts to mimic natural growth. The medium needs to be analyzed by untargeted metabolomics (mass spectrometry and/or nuclear magnetic resonance) over multiple time points to provide insight into the presence and abundance of specific metabolites. Metabolites that strongly change in abundance during pathogen growth and between subsequent sampling stages are likely assimilated or secreted. This can be indicative of a nutrient transporter on the plasma membrane that is capable of transporting the respective metabolite. In addition, isotope-labeled metabolites can be added to the medium to test the assimilation of specific nutrients (Ah-Fong et al., 2019), such as carbohydrates and lipids. For metabolites for which changes in abundance are measured, uptake and demand (transport) reactions should be added to the model.

Fourth, the relative pathogen biomass composition and growth rates should be measured. A promising approach for this is Fourier-transform infrared spectroscopy (FTIR; Mayers et al., 2013). This method was optimized for analyses of brown algae and was successfully used in the reconstruction of a GEM for the diatom *Phaeodactylum tricornutum* to quantify the percentages of carbohydrate, protein, DNA/RNA, and fatty acids per gram of cellular dry weight (Levering et al., 2016). Once the main classes of biomass components are quantified and are related to growth rate, more specific metabolites can be assigned based on traditional metabolomics methods (e.g., chromatography and mass spectrometry) and incorporated into the GEM.

Fifth, predicted phenotypes, e.g., induced by specific nutrient starvation, should be validated by growth experiments and gene/reaction essentiality should be validated in knockout or knockdown mutants, for instance, as was done for the nitrate assimilation cluster (Abrahamian et al., 2016). As discussed, we anticipate that CRISPR-Cas gene editing will be successfully employed in many pathogens in the coming years, but as an alternative, gene silencing mutants can be generated to investigate metabolic perturbations. The model should be updated with novel findings, and discrepancies should lead to corrections of the model. For instance, when the knockout of a predicted essential gene is not lethal *in vitro*, there are likely alternative enzymes or metabolic routes that compensate for this mutation. The model should be inspected on incorrect annotations or missing reactions accordingly.

Sixth, the refined pathogen GEM should be integrated with a similarly refined GEM for its host. This necessitates deploying more sophisticated constraints and objective functions to simulate a more realistic symbiosis for this pathosystem, such as multi-objective simulations to address the competition for nutrients (Jamshidi and Raghunathan, 2015).

In addition to the here proposed steps, there is a large and rapidly increasing number of methods and algorithms that can be applied to GEMs to gain further insights into the complex system of pathogen–host interactions (Lewis et al., 2012). For instance, regulatory networks could be inferred from (anti-)correlated expression patterns in dual RNA-Seq data and other experimental data and integrated into GEMs to further constrain the fluxes, in order to learn how metabolism is regulated during infection (Peyraud et al., 2018). Collectively, we anticipate that these steps will lead to high-quality pathogen and pathogen–host GEMs that can be used to identify novel targets for disease control and further help to understand how pathogens interact with their hosts.

## Conclusion

Systems biology, in particular GEMs, offers a unique approach to study oomycetes and their intricate interactions with their hosts. GEMs not only offer a holistic overview of metabolism, but also constitute a foundation on which to incorporate omics measurements at various levels, allowing integrated analyses of key processes in pathogenesis and pathogen–host interaction. Although there are still a number of remaining technical and methodological challenges, GEMs hold great promise for providing mechanistic insight into strategies exploited by oomycetes to proliferate and infect their hosts, ultimately allowing us to develop new means of controlling these highly relevant pathogens.

## Author Contributions

SR wrote the first draft of the manuscript. FG edited sections of the manuscript. All authors contributed to manuscript revision, read, and approved the submitted version.

## Funding

This work was funded by the Food-for-Thought campaign from Wageningen University Fund.

## Conflict of Interest

The authors declare that the research was conducted in the absence of any commercial or financial relationships that could be construed as a potential conflict of interest.

## Publisher’s Note

All claims expressed in this article are solely those of the authors and do not necessarily represent those of their affiliated organizations, or those of the publisher, the editors and the reviewers. Any product that may be evaluated in this article, or claim that may be made by its manufacturer, is not guaranteed or endorsed by the publisher.
